# AnthWest, occurrence records for wool carder bees of the genus *Anthidium* (Hymenoptera, Megachilidae, Anthidiini) in the Western Hemisphere

**DOI:** 10.3897/zookeys.408.5633

**Published:** 2014-05-12

**Authors:** Terry Griswold, Victor H. Gonzalez, Harold Ikerd

**Affiliations:** 1 USDA-ARS. Bee Biology & Systematics Laboratory, Utah State University, Logan, Utah 84322-5310, USA; 2 Southwestern Oklahoma State University, Biological Sciences, 100 Campus Drive, Weatherford, Oklahoma, 73096, USA

**Keywords:** Anthophila, Apoidea, bees, invasive species, North America, South America, pollinators, biodiversity, floral hosts

## Abstract

This paper describes AnthWest, a large dataset that represents one of the outcomes of a comprehensive, broadly comparative study on the diversity, biology, biogeography, and evolution of *Anthidium* Fabricius in the Western Hemisphere. In this dataset a total of 22,648 adult occurrence records comprising 9657 unique events are documented for 92 species of *Anthidium*, including the invasive range of two introduced species from Eurasia, *A. oblongatum* (Illiger) and *A. manicatum* (Linnaeus). The geospatial coverage of the dataset extends from northern Canada and Alaska to southern Argentina, and from below sea level in Death Valley, California, USA, to 4700 m a.s.l. in Tucumán, Argentina. The majority of records in the dataset correspond to information recorded from individual specimens examined by the authors during this project and deposited in 60 biodiversity collections located in Africa, Europe, North and South America. A fraction (4.8%) of the occurrence records were taken from the literature, largely California records from a taxonomic treatment with some additional records for the two introduced species. The temporal scale of the dataset represents collection events recorded between 1886 and 2012. The dataset was developed employing SQL server 2008 r2. For each specimen, the following information is generally provided: scientific name including identification qualifier when species status is uncertain (*e.g.* “Questionable Determination” for 0.4% of the specimens), sex, temporal and geospatial details, coordinates, data collector, host plants, associated organisms, name of identifier, historic identification, historic identifier, taxonomic value (*i.e.*, type specimen, voucher, etc.), and repository. For a small portion of the database records, bees associated with threatened or endangered plants (~ 0.08% of total records) as well as specimens collected as part of unpublished biological inventories (~17%), georeferencing is presented only to nearest degree and the information on floral host, locality, elevation, month, and day has been withheld. This database can potentially be used in species distribution and niche modeling studies, as well as in assessments of pollinator status and pollination services. For native pollinators, this large dataset of occurrence records is the first to be simultaneously developed during a species-level systematic study.

## Project details

**Project title:** Wool carder bees of the genus *Anthidium* (Hymenoptera: Megachilidae, Anthidiini) in the Western Hemisphere

**Personnel:** Terry Griswold (author), Victor H. Gonzalez (author), Harold Ikerd (database manager, author).

**Funding:** National Science Foundation grants DEB-0742998 and DBI-0956388.

**Figure 1.**
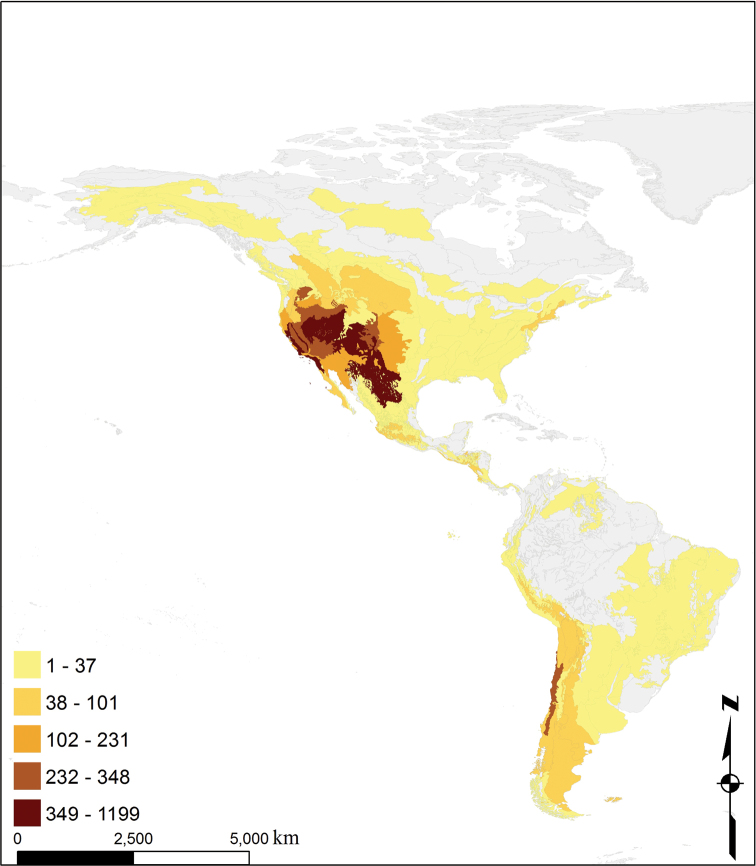
Collecting intensity of *Anthidium* by ecoregion in the Western Hemisphere. Number of collection events defined as unique date and latitude and longitude combinations per each WWF ecoregion (Olson et al. 2001). The 10 ecoregions with the highest number of events were: Great Basin shrub steppe, 1199; California coastal sage and chaparral, 930; Colorado Plateau shrublands, 782; California interior chaparral and woodlands, 768; Chihuahuan desert, 648; Mojave desert, 348; Sierra Nevada forests, 317; Chilean matorral, 314; Colorado Rockies forests, 303; and California Central Valley grasslands, 296.

**Figure 2.**
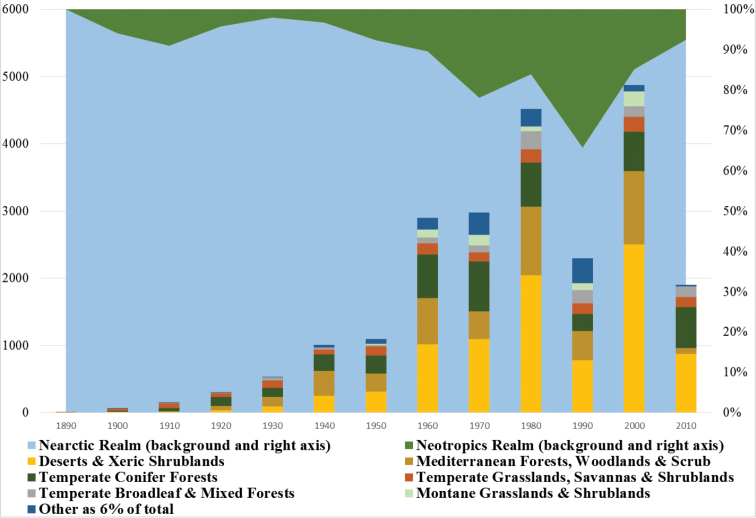
Collection intensity across decades by WWF Biomes and Realms. The following biomes comprised less than 3% each and were combined: Tropical and Subtropical Coniferous Forests, Tropical and Subtropical Dry Broadleaf Forests, Tropical and Subtropical Moist Broadleaf Forests, Tropical and Subtropical Grasslands, Savannas and Shrublands, Mangroves, Boreal Forests/Taiga, Rock and Ice, Tundra, and Flooded Grasslands and Savannas.

**Study area description:** The database covers a wide range of ecosystems found in both North and South America, from -62° to 79° in latitude and -174° to -22° in longitude. A large portion of the records in North America are from xeric regions (Great Basin, Colorado Plateau, Mojave, Sonoran, and Chihuahuan Deserts) and Mediterranean California, while those from South America are mostly from the xeric regions on the flanks of the Andes (Figs 1, 2). No records for *Anthidium* are known from the Caribbean islands. Much of the data set comes from general bee collecting. Additional material in western United States comes from multi-year intensive, systematic bee faunal studies in protected landscapes.

While the majority of species of *Anthidium* occupy a small number of ecoregions (< 5), some species such as *Anthidium tenuiflorae* Cockerell are widespread, occurring in as many as 41 ecoregions. Many *Anthidium* have distributions that include critical, endangered, or vulnerable, as well as relatively stable or intact, ecoregions (Table 1) based on WWF (World Wild Fund for Nature) designations (Olson and Dinerstein 2002). Known distributions for 16 species are largely or entirely within critical or endangered ecoregions with at least 90% of collection records from such designated areas. An additional 22 species had at least 90% of collection records from within vulnerable ecoregions. Few native *Anthidium* spanned both Nearctic and Neotropic Realms (8.8%).

**Table 1.** Distribution and relative abundance of species of *Anthidium* by ecoregions in the Western Hemisphere. For a particular species, abundance on WWF designated ecoregion status was estimated as the percentage of specimen records occurring in those ecoregions over the total number of specimen records of that species.

Species	# of Ecoregions	# of specimens	Ecoregion Status		
			Critical or Endangered (%)	Vulnerable (%)	Relatively Stable or Intact (%)
*Anthidium adelphum*	3	48	2	2	96
*Anthidium adriani*	3	19	100	0	0
*Anthidium alsinai*	1	1	0	100	0
*Anthidium andinum*	4	10	20	80	0
*Anthidium anurospilum*	3	22	0	18	82
*Anthidium atacamense*	4	18	6	17	78
*Anthidium atrifrons*	26	985	42	19	39
*Anthidium atripes*	16	471	6	38	56
*Anthidium atripoides*	3	127	0	0	100
*Anthidium aymara*	4	13	23	69	8
*Anthidium aztecum*	3	11	100	0	0
*Anthidium banningense*	19	370	65	13	22
*Anthidium cafayate*	3	4	0	100	0
*Anthidium calchaqui*	3	8	0	100	0
*Anthidium chamelense*	4	17	100	0	0
*Anthidium chilense*	8	947	96	1	3
*Anthidium chubuti*	6	15	67	0	33
*Anthidium clypeodentatum*	25	177	45	18	37
*Anthidium cochimi*	11	88	7	47	47
*Anthidium cockerelli*	13	1169	1	6	93
*Anthidium collectum*	16	730	47	50	4
*Anthidium colliguayanum*	3	54	96	0	4
*Anthidium cuzcoense*	2	8	88	13	0
*Anthidium dammersi*	7	333	0	4	95
*Anthidium danieli*	2	8	88	0	13
*Anthidium danunciae*	1	5	0	100	0
*Anthidium decaspilum*	5	34	24	3	74
*Anthidium deceptum*	5	112	0	86	14
*Anthidium duomarginatum*	6	210	8	0	92
*Anthidium edwardsii*	15	369	63	31	6
*Anthidium edwini*	3	13	92	0	8
*Anthidium emarginatum*	14	505	40	7	53
*Anthidium espinosai*	5	47	15	2	83
*Anthidium formosum*	19	167	51	15	34
*Anthidium friesei*	8	183	4	91	4
*Anthidium funereum*	12	174	15	39	46
*Anthidium gayi*	7	502	93	2	5
*Anthidium hallinani*	9	137	93	7	0
*Anthidium igori*	1	5	0	100	0
*Anthidium illustre*	16	539	42	42	16
*Anthidium insignissimum*	2	13	31	69	0
*Anthidium jocosum*	14	422	15	15	69
*Anthidium kolla*	2	9	0	100	0
*Anthidium labergei*	2	38	0	95	5
*Anthidium larocai*	1	1	0	100	0
*Anthidium latum*	5	11	27	73	0
*Anthidium luizae*	1	1	0	100	0
*Anthidium maculifrons*	39	522	80	19	1
*Anthidium maculosum*	35	1356	55	24	22
*Anthidium macushi*	4	32	0	94	6
*Anthidium manicatum*	23	635	53	3	44
*Anthidium mapuche*	4	44	89	0	11
*Anthidium masunariae*	1	2	100	0	0
*Anthidium meloi*	1	5	0	100	0
*Anthidium michenerorum*	4	16	94	0	6
*Anthidium mormonum*	32	1612	51	15	35
*Anthidium multispinosum*	1	1	0	100	0
*Anthidium neffi*	1	1	100	0	0
*Anthidium nigerrimum*	4	6	17	50	33
*Anthidium oblongatum*	7	163	96	4	0
*Anthidium paitense*	1	7	0	100	0
*Anthidium pallidiclypeum*	8	181	14	34	52
*Anthidium palliventre*	9	396	66	32	2
*Anthidium palmarum*	17	984	7	12	81
*Anthidium parkeri*	11	187	93	7	0
*Anthidium paroselae*	9	563	0	3	97
*Anthidium penai*	2	26	100	0	0
*Anthidium peruvianum*	3	23	0	91	9
*Anthidium placitum*	22	1034	30	19	51
*Anthidium platyfrons*	1	3	0	0	100
*Anthidium porterae*	18	982	41	31	27
*Anthidium psoraleae*	7	17	88	0	12
*Anthidium quetzalcoatli*	7	38	95	0	5
*Anthidium rafaeli*	2	9	0	100	0
*Anthidium rodecki*	6	411	21	1	79
*Anthidium rodriguezi*	14	74	96	4	0
*Anthidium rozeni*	1	1	0	100	0
*Anthidium rubripes*	11	76	9	70	21
*Anthidium sanguinicaudum*	4	8	13	88	0
*Anthidium schwarzi*	9	104	12	61	28
*Anthidium sertanicola*	1	1	0	100	0
*Anthidium sonorense*	10	77	3	12	86
*Anthidium sparsipunctatum*	4	90	3	97	0
*Anthidium spatulatum*	2	41	2	0	98
*Anthidium tarsoi*	1	2	0	100	0
*Anthidium tenuiflorae*	41	1189	37	22	42
*Anthidium toro*	2	65	0	22	78
*Anthidium utahense*	29	2409	39	49	12
*Anthidium vigintiduopunctatum*	9	41	24	76	0
*Anthidium vigintipunctatum*	4	30	3	97	0
*Anthidium weyrauchi*	1	11	0	100	0

**Figure 3.**
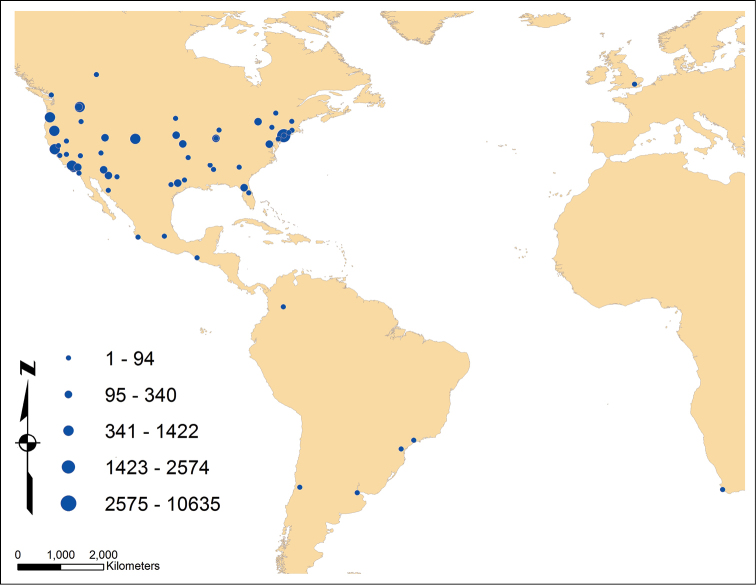
Location of 60 insect collections with number of specimens of *Anthidium* examined.

**Design description:** The purpose of this dataset is to make available data associated with bees of the genus *Anthidium* in the Western Hemisphere. The dataset was developed during the course of a species-level revision of the genus (Gonzalez and Griswold 2013). Most records come from specimens deposited in the first author’s host institution or acquired on loan from multiple bee depositories, primarily in North America, but some from South American and European institutions (Fig. 3). Permitting issues limited access to some South American institutions. All such specimens were identified by V.H. Gonzalez and/or T. Griswold. Additional California records from Grigarick and Stange (1968) were captured for all species whose taxonomic concept was not modified in Gonzalez and Griswold (2013). Subsequent to identification, individual specimens were processed by a team of assistants at the USDA-ARS Bee Biology & Systematics Laboratory (BBSL). Individual specimens were entered into the US National Pollinating Insects Database (USNPID) using data entry forms where each specimen received a unique identifier (see below). These forms used authority files for bees, locations, collectors and plants. Where locations were not already georeferenced in the database they were georeferenced using Google Earth^tm^ (http://earth.google.com/) or GEOlocate (http://www.museum.tulane.edu/geolocate/). Georeferencing used the form of decimal latitude and longitude in the WGS84 datum. Where georeferencing in the form of UTMs; township, range and section; or degree-minute-seconds was present on the specimen label, these were transformed, but the original label georeferencing was captured in the location authority files. Records were analyzed geospatially using ArcGIS and WWF Biotic Regions. Twenty-two records (<0.1%) were excluded from biotic regions analysis due to questionable identification and/or label data.

Databasing processes for the USNPID have evolved over the 25 years since initiation. Processing, originally considered as too costly, has since been incorporated into the databasing process. Verbatim label data capture originally only for holotypes, was expanded first to loaned specimens and now to all retro-active data capture. When validity of entry fields is questioned, verbatim information is queried before pulling the specimen from the collection, saving both time and potential handling hazards. Addition of that tracking data (*e.g.* date of record entry, date of record modification, logging of entry person) and use of authority tables were essential to data quality, yet amounted to negligible additional data capture costs.

**The data underpinning the analysis reported in this paper are deposited at** GBIF, the Global Biodiversity Information Facility, http://ipt.pensoft.net/ipt/resource.do?r=anthidium.

## Taxonomic coverage

**Figure 4.**
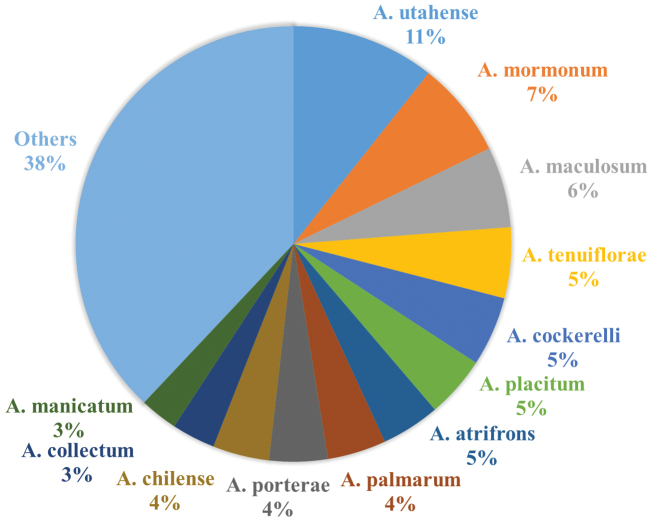
Percentage of specimen records per species of *Anthidium* in the AnthWest database. The category “Others” correspond to remaining species with specimen records accounting for less than 2%. All individual species shown except *Anthidium chilense* are Nearctic.

**General taxonomic coverage description:** The coverage of this dataset includes all 92 species of the bee genus *Anthidium* known to occur in the Western Hemisphere, including two that are introduced. *Anthidium* belongs to the tribe Anthidiini and is among the most diverse genera of the family Megachilidae. Based on the materials used in nest construction, anthidiines are broadly classed into two groups, carder bees and resin bees. While resin bees are generically diverse in the Western Hemisphere, *Anthidium* is the sole representative of carder bees in the Americas. As such this dataset documents all of a functional bee group for the Americas. The greatest number of data records are for two widespread western North American species, *Anthidium utahense* Swenk (2409 records) and *Anthidium mormonum* Cresson (1615 records) (Fig. 4). The species with the least number of records are *Anthidium alsinai* Urban, *Anthidium isabelae* Urban, *Anthidium larocai* Urban, *Anthidium luizae* Urban, *Anthidium multispinosum* Gonzalez & Griswold, *Anthidium neffi* Gonzalez & Griswold, and *Anthidium rozeni* Urban, each represented by a single data record. Though these species are rare in collections, there is no knowledge whether they are rare in nature, though at least for *Anthidium multispinosum*, it is likely that it has a restricted distribution. No *Anthidium* in the Western Hemisphere have formally been listed as threatened or endangered.

*Anthidium* are occasionally associated with rare, threaten or endangered plants. Only a handful of such associations with state and/or federally listed plant bee records are included in the dataset (Table 2). Published records provide georeference only to the nearest degree, and floral host, month and day fields will have information hidden.

**Table 2.** Species of *Anthidium* of the Western Hemisphere recorded from rare, threaten or endangered plants.

Plant species	Bee species	# of records
Asteraceae		
*Erigeron rhizomatus* Cronquist	*Anthidium mormonum*	3
	*Anthidium duomarginatum*	3
	*Anthidium maculosum*	1
Cactaceae		
*Sclerocactus mesae-verdae* (Boissev. ex Hill & Salisb.) L.D. Benson	*Anthidium emarginatum*	1
*Pediocactus sileri* (Boissev. ex Hill & Salisb.) L.D. Benson	*Anthidium emarginatum*	1
Fabaceae		
*Astragalus humillimus* A. Gray	*Anthidium dammersi*	2
*Dalea formosa* Torr.	*Anthidium palmarum*	7

All specimens in this dataset have been reviewed by the authors or are easily determined taxa that have been reviewed by experts in bee taxonomy (*e.g.*, John Ascher, for some AMNH material; A. A. Grigarick and L. A. Stange for California records in Grigarick and Stange 1968). Records with questionable determinations, label information or data have been withheld.

### Taxonomic ranks

**Kingdom:** Animalia

**Phylum:** Arthropoda

**Class:** Insecta

**Order:** Hymenoptera

**Family:** Megachilidae

**Genus:** *Anthidium*

**Common names:** wool carder bees

## Spatial coverage

**General spatial coverage:** This dataset includes species occurrences of bees in the genus *Anthidium* across the entire Western Hemisphere, from Alaska to southern Chile and southern Argentina, and from below sea level in Death Valley, California, USA, to 4700 m a.s.l. in Tucumán, Argentina. Within North America coverage is most complete for temperate regions, though diminishing diversity correlated with declining latitude cannot be ignored.

### Coordinates

62° to 79° latitude and -174° to -22° longitude

## Temporal coverage

Records in AnthWest span more than a century, from May 1886 to February 2012. The majority of the records are from the past four decades (Fig. 2). In temperate North America, here restricted to Canada and the United States, *Anthidium* is most active during the late spring and summer months; the majority of the records are for May through August. In alpine regions (> 3000m) the season is narrowed to May through September, but largely June through August, peaking in July.

## Datasets

**Dataset description:** AnthWest is a result of a broadly comparative study on the diversity, biology, biogeography, and evolution of bees in the genus *Anthidium* in the Western Hemisphere. The dataset includes 22,648 occurrence records for 92 species of *Anthidium*, including two introduced species from Eurasia. Each record consists of the species name, locality, collector’s name, collection date, latitude, longitude, host plants, associated organisms, name of identifier, taxonomic value (*i.e.*, type specimen, voucher, etc.), and repository. When coordinates for collection sites were not provided on the label, they were extracted using Google Earth^tm^ (http://earth.google.com/) or GEOlocate (http://www.museum.tulane.edu/geolocate/). To guarantee the high quality of the data, most records in the dataset correspond to individual specimens examined by the authors during this project, representing 60 biodiversity collections in Europe, Africa, North and South America (Fig. 3). A small fraction (4.8%) of the occurrence records were extracted from the literature. Only literature records for which there was a high degree of certainty in the identification were included. The vast majority of these published records were taken from the rigorous study of California Anthidiini by Grigarick and Stange (1968). Their records were included for all *Anthidium* species except *Anthidium atripes* and *Anthidium emarginatum*, which in Gonzalez and Griswold (2013) are recognized as species complexes. The balance, 30 records of the introduced *Anthidium manicatum* and *Anthidium oblongatum* (Miller et al. 2002, Maier 2009, Tonietto and Ascher 2008), were included because these are distinctive species that could not be confused with any native species nor with each other.

**Figure 5.**
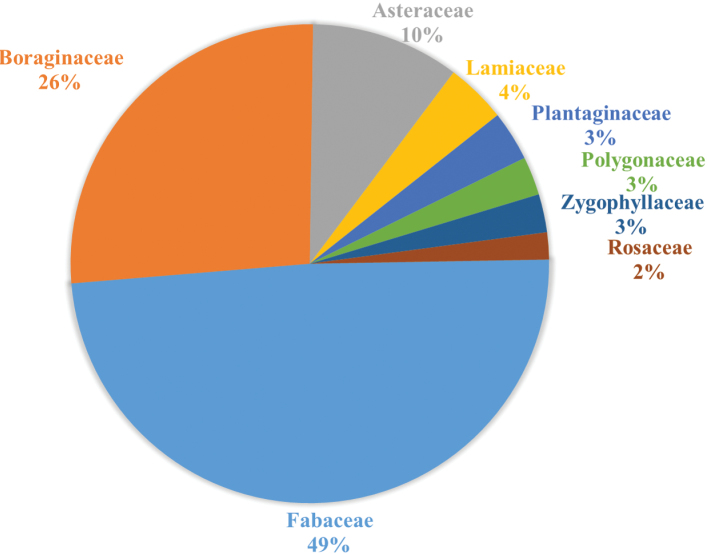
Plant families visited by *Anthidium* in the Western Hemisphere. Only families represented by at least 2% of the total 5358 floral visitation records in the database are shown.

As with most other bees, floral resources are essential for reproductive success of *Anthidium*. Floral records indicate a broad array of floral visitation based on the quarter (24%) of AnthWest records that include floral visits. While visitation includes 56 plant families and over 100 species, Fabaceae and Boraginaceae dominated the dataset, together accounting for 75% of the records (Fig. 5).

**Figure 6.**
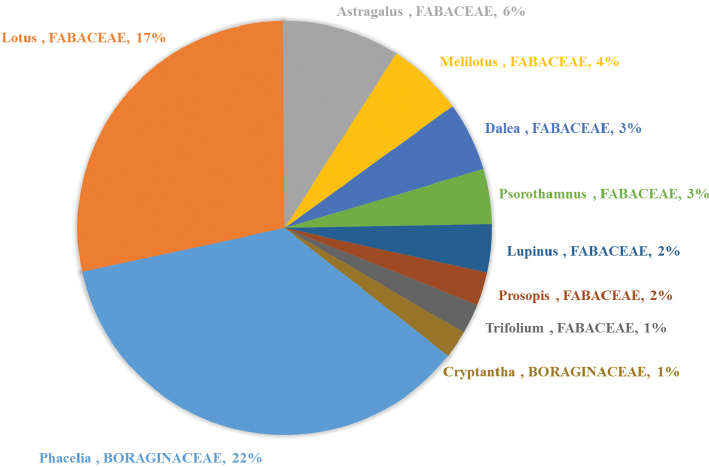
Percentage of plant records for the ten most visited plant genera (*n* = 5358 floral visitation records).

Analysis of plant records at the generic level similarly shows the dominance of Fabaceae and Boraginaceae; all top ten floral associations belong to these two families, but *Phacelia*, the most visited genus belongs not to Fabaceae but to Boraginaceae (Fig. 6).

Records for 34 name-bearing types of *Anthidium* are also included in the database.

**Study extent:** Because this dataset was developed as part of research that was focused on taxonomic revisionary work, sampling was not the focus of efforts; rather the data represents the aggregate of what we know about the distribution and behavior of *Anthidium* from existing material. Carder bees are diurnal, and are only active when temperatures are well above freezing and only during the growing season when floral resources are potentially available.

**Sampling description:** Specimen records captured in AnthWest are the result of: 1) non-systematic collections usually as part of general entomological collecting events or ones focused on bees in general; 2) standardized biodiversity surveys conducted by the USDA Pollinating Insects Research Unit using a combination of net and pan traps; 3) trap nest studies; and 4) specimens resulting from studies on pollination and reproductive biology of threatened or endangered plants.

**Figure 7.**
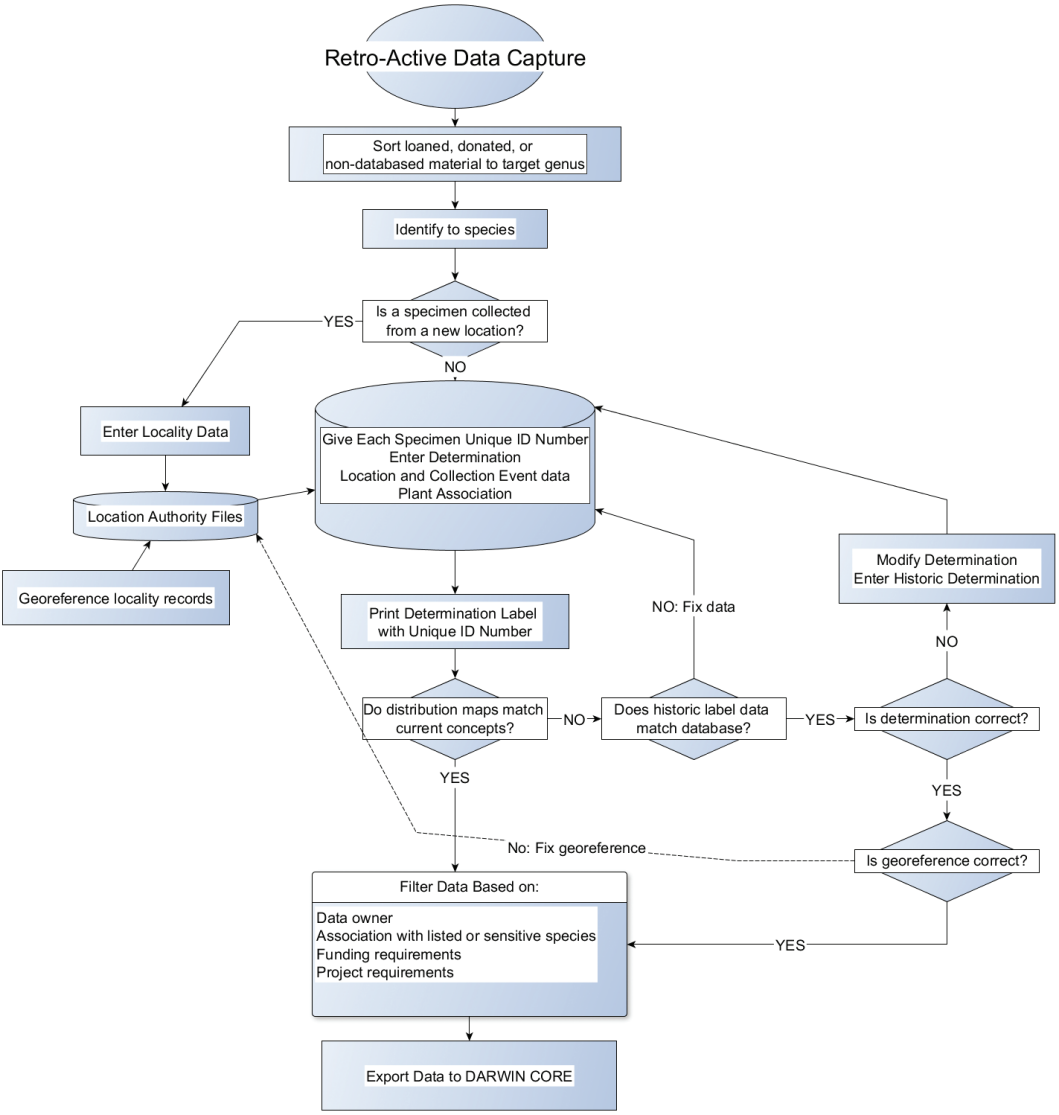
Flow chart for retroactive data capture of museum specimens.

**Figure 8.**
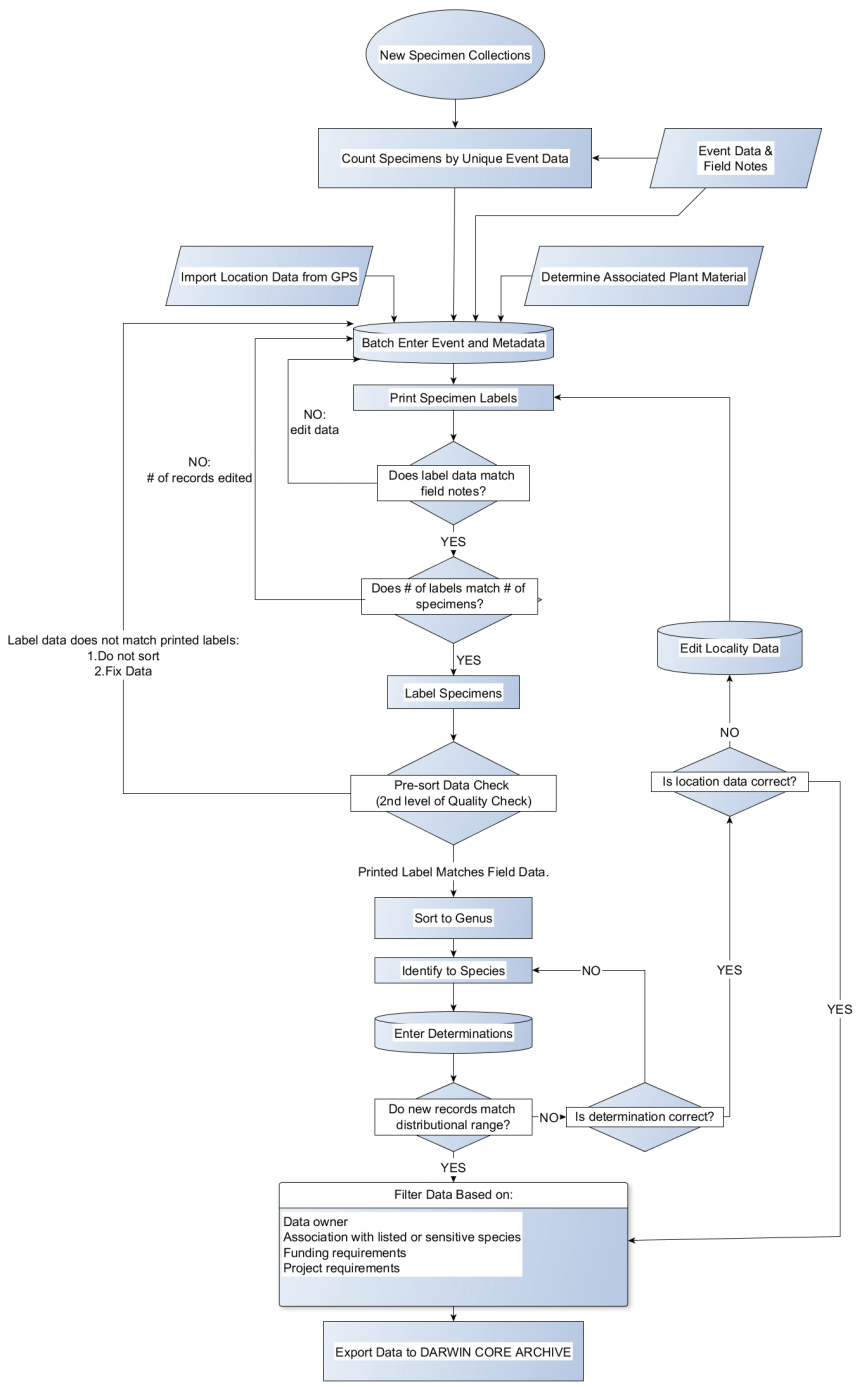
Flow chart for processing of new specimen samples.

**Quality control:** All individual specimens included in this dataset were examined during the course of the taxonomic revision using distribution maps and raw data following standardized protocols (Figs 7, 8). Records with questionable data on original insect labels were included in the dataset but distinguishable by notes in the DWC field “Identification Qualifier”. These records were excluded from published distribution maps in the species-level revision of the genus (Gonzalez and Griswold 2013). A small fraction (4.8%) of the occurrence records were taken from the literature (see above), largely California records from a taxonomic treatment with some additional records for the two introduced species (*Anthidium manicatum* and *Anthidium oblongatum*). These records are highlighted in the Darwin Core [DWC] fields “Associated References” and “Occurrence Remarks” as well as a denoted with a “PUB” prefix in the catalog number.

**Step description:** Two separate work flows were employed for data capture, which differed fundamentally on where in the process material was determined by the revisionary authors. Retroactive data capture (Fig. 7) incorporated loaned specimens, publication records, and previously non-databased specimens in the U.S. National Pollinating Insects Collection, all of which follows after the identification process. Publication records were treated similarly to retroactive data capture except each record represents a summation of males and females with identical collecting event data. Beginning in 2005, new specimen records (Fig. 8) were batch entered into the database for projects and opportunistic collection events alike. Specimen identification and subsequent update to the database occurred after record and event metadata had been entered. New specimen collections also had a work flow that resulted in a greater number of data quality checks by technicians and primary researchers.

**Purpose:** The purpose of this dataset is to make available data associated with bees of the genus *Anthidium* in the Western Hemisphere. The dataset was developed during the course of a species-level revision of the genus (Gonzalez and Griswold 2013). This dataset can potentially be used in species distribution and niche modeling studies, as well as in assessments of pollinator status and pollination services.

**IP Rights: Licenses of use:** This work is licensed under a Creative Commons Attribution-NonCommercial-ShareAlike 3.0 Unported License. http://creativecommons.org/licenses/by-nc-sa/3.0/ Records highlighted in the Darwin Core [DWC] fields “rights” and “rightsholder” indicate specimens that have addition usage rights.

**Collection Data:** For all collections, including those not listed in the Global Registry of Biodiversity Repositories (www.grbio.org) the Institution code listed below is included in the DWC field “owner Institution Code”.

**AMNH** American Museum of Natural History, New York, New York, USA [2574 records]

**ARDU** M. Arduser, Missouri Department of Conservation, St. Charles, Missouri [32 records]

**ASUT** Arizona State University, Frank M.H. Natural History Museum, Tempe, Arizona, USA [145 records]

**BBNP** Big Bend National Park, Big Bend, Texas, USA [7 records]

**BBSL** USDA-ARS Bee Biology and Systematic Laboratory, Logan, Utah, USA [11123 records]

**BNHM** British Natural History Museum, London, UK [19 records]

**BYUC** Monte L. Bean Life Science Museum, Arthropod Collection, Provo, Utah, USA [105 records]

**CAES** Connecticut Agriculture Experiment Station, New Haven, Connecticut, USA [30 records]

**CAS** California Academy of Sciences, San Francisco, California, USA [637 records]

**CEET** Colección de Insectos Asociados a Plantas Cultivadas en la Frontera Sur, El Colegio de la Frontera Sur, Tapachula, Chiapas, Mexico [1 record]

**CIDA** College of Idaho, Museum of Natural History, Caldwell, Idaho, USA [25 records]

**CNC** Canadian National Collection of Insects, Arachnids & Nematodes, Ottawa, Ontario, Canada [5 records]

**CTMI** Central Texas Melittological Institute, Austin, Texas, USA [34 records]

**CUIC** Cornell University Insect Collection, Ithaca, New York, USA [33 records]

**DEVA** Death Valley National Park, Furnace Creek, California, USA [11 records]

**DZUP** Departamento de Zoologia, Universidade Federal do Paraná, Curitiba, Brazil [16 records]

**EBCC** Estación de Biología Chamela, Universidad Nacional Autónoma de Mexico, San Patricio, Jalisco, Mexico [24 records]

**EMEC** Essig Museum of Entomology, University of California, Berkeley, California, USA [1173 records]

**FCDA** Fresno County Department of Agriculture, Fresno, California, USA [3 records]

**FMNH** Field Museum of Natural History, Chicago, Illinois, USA [7 records]

**FSCA** Florida State Collection of Arthropods, Florida State University, Gainesville, Florida, USA [122 records]

**GSENM** Grand Staircase-Escalante National Monument, Kanab, Utah, USA [12 records]

**HNH_ent** Dartmouth College, Hanover, New Hampshire, USA [1 record]

**INHS** Illinois Natural History Survey, Urbana, Illinois, USA [161 records]

**LACM** Natural History Museum of Los Angeles County, Los Angeles, California, USA [1422 records]

**MACN** Museo Argentino de Ciencias Naturales ‘Bernardino Rivadavia’, Buenos Aires, Argentina [36 records]

**MEM** Mississippi Entomological Museum, Mississippi State University, Starkville, Mississippi, USA [6 records]

**MEUC** Colección del Museo Entomológico Luis Peña, Departamento de Sanidad Vegetal, Universidad de Chile, Santiago, Chile [1 record]

**SS; RR** Snelling and G.I. Stage personal collections; USA [9 records]

**MZUSP** Museu de Zoologia, Universidade de São Paulo, São Paulo, Brazil [4 records]

**NMNH** Smithsonian National Museum of Natural History, Washington, D.C., USA [340 records]

**NVDA** Nevada State Department of Agriculture, Reno, Nevada, USA [17 records]

**NYBG** New York Botanical Garden, New York, New York, USA [1 record]

**OSAC** Oregon State Arthropod Collection, Corvallis, Oregon, USA [580 records]

**PCYU** Packer’s Apoidea Collection at York University, Toronto, Ontario, Canada [239 records]

**PHIL** University of the Sciences in Philadelphia, Philadelphia, Pennsylvania, USA [32 records]

**PINN** Pinnacles National Monument, Paicines, California, USA [6 records]

**PMAE** Royal Alberta Museum, Edmonton, Alberta, Canada [12 records]

**RUDZ** Rhodes University, Grahamstown, South Africa, [44 records]

**SDNHM** San Diego Natural History Museum, San Diego, California, USA [94 records]

**SDSU** Severin-McDaniel Insect Collection, South Dakota State University, Brookings, South Dakota, USA [48 records]

**SEMC** Snow Entomological Museum, University of Kansas, Lawrence, Kansas, USA [246 records]

**SFUC** Simon Fraser University, Burnaby, British Columbia, Canada [1 record]

**SWRS** Southwestern Research Station, Portal, Arizona, USA [7 records]

**TAMU** Texas A&M University Insect Collection, College Station, Texas, USA [101 records]

**UAAM** University of Arkansas Arthropod Museum, Fayetteville, Arkansas, USA [4 records]

**UAIC** University of Arizona Insect Collection, Tucson, Arizona, USA [150 records]

**UCDC** R.M. Bohart Museum of Entomology, University of California, Davis, California, USA [658 records]

**UCF** University of Central Florida Collection of Arthropods, Department of Biology, Orlando, Florida, USA [61 Records]

**UCMC** University of Colorado Museum of Natural History, Boulder, Colorado, USA [762 records]

**UCMS** University of Connecticut, Storrs, Connecticut, USA [38 records]

**UCR** University of California, Riverside, California, USA [298 records]

**UGCA** University of Georgia, Athens, Georgia, USA [68 records]

**UNAB** Museo Entomológico, Departamento de Agronomía, Universidad Nacional de Colombia, Bogotá, Colombia [1 record]

**UNAM** Museo de Zoología Alfonso L. Herrera, Facultad de Ciencias, Universidad Nacional Autónoma de México, Mexico [33 records]

**UNSM** University of Nebraska State Museum, Lincoln, Nebraska, USA [111 records]

**USON** Universidad de Sonora, Hermosillo, Sonora, Mexico [1 record]

**WFBM** W.F. Barr Entomological Collection, University of Idaho, Moscow, Idaho, USA [639 records]

**WSU** Maurice T. James Entomological Collection, Washington State University, Pullman, Washington, USA [110 records]

**ZAVOR** Zavortink Private Collection, Davis, California, USA [14 records]

**Specimen preservation method and curatorial units:** Records represent pinned, dried adult individuals with attached label data stored in most cases in standard insect museum drawers preserved from dermestid damage by routine freezing of drawers at -20 C. Reviewed *Anthidium* specimens followed the basic process for Hymenoptera preservation and labeling outlined in Huber (1998). Newly collected BBSL specimens are given catalog numbers during initial labeling. Material sent for identification and loans were given unique catalog numbers after final identification and data entry.

**Object name:** Darwin Core Archive Wool carder bees of the genus *Anthidium* in the Western Hemisphere

**Character encoding:** UTF-8

**Format name:** Darwin Core Archive format

**Format version:** 1.0

**Distribution:** http://ipt.pensoft.net/ipt/archive.do?r=anthidium

**Publication date of data:** 2013-03-25

**Language:** English

**Licenses of use:** The U.S. National Pollinating Insects Database [United States Department of Agriculture, Agriculture Research Service, Bee Biology and Systematics Laboratory, Logan, Utah] is made available under the Open Database License: http://opendatacommons.org/licenses/odbl/1.0/. Any rights in individual contents of the database are licensed under the Database Contents License: http://opendatacommons.org/licenses/dbcl/1.0/.

**Metadata language:** English

**Date of metadata creation:** 2012-06-27

**Hierarchy level:** Dataset

## Additional information

We are greatly indebted to each of the curators, collection managers, and staff from the collections that we visited, or from which we borrowed specimens for this study. This work would not have been possible without their constant and valuable support. The names of the institutions and their personnel are indicated in the section of Material and Methods of Gonzalez and Griswold (2013). Anonymous reviewers provided insightful comments and suggestions that improved this manuscript. This study was supported in part by National Science Foundation grants DEB-0742998 and DBI-0956388.

## References

[B2] Gonzalez VH Griswold T (2013) Wool carder bees of the genus *Anthidium* in the Western Hemisphere (Hymenoptera: Megachilidae): diversity, host plant associations, phylogeny, and biogeography. Zoological Journal of the Linnean Society 168: 221-425. doi: 10.1111/zoj.12017

[B3] Grigarick AA Stange LA (1968) The pollen-collecting bees of the Anthidiini of California. Bulletin of the California Insect Survey 9: 1-113

[B4] Huber JT (1998) The importance of voucher specimens, with practical guidelines for preserving specimens of the major invertebrate phyla for identification. Journal of Natural History 32(3): 367-385. doi: 10.1080/00222939800770191

[B5] Miller SR Gaebel R Mitchell RJ Arduser M (2002) Occurrence of two species of Old World bees, *Anthidium manicatum* and *A. oblongatum* (Apoidea: Megachilidae), in northern Ohio and southern Michigan. The Great Lakes Entomologist 35(1): 65-69

[B6] Maier CT (2009) New distributional records of three alien species of Megachilidae (Hymenoptera) from Connecticut and nearby states. Proceedings of the Entomological Society of Washington 111: 775-784. doi: 10.4289/0013-8797-111.4.775

[B7] Olson DM Dinerstein E (2002) The Global 200: Priority ecoregions for global conservation. Annals of the Missouri Botanical Garden 89(2): 199-224. doi: 10.2307/3298564

[B8] Olson DM Dinerstein E Wikramanayake ED Burgess ND Powell GVN Underwood EC D’Amico JA Itoua I Strand HE Morrison JC Loucks CJ Allnutt TF Ricketts TH Kura Y Lamoreux JF Wettengel WW Hedao P Kassem KR (2001) Terrestrial ecoregions of the world: a new map of life on Earth. Bioscience 51(11): 933-938

[B9] Tonietto RK Ascher JS (2008) Occurrence of the old world bee species *Hylaeus hyalinatus*, *Anthidium manicatum*, *A. oblongatum*, and *Megachile sculpturalis*, and the native species *Coelioxys banksi*, *Lasioglossum michiganense*, and *L. zophops* in Illinois (Hymenoptera: Apoidea: Colletidae, Halictidae, Megachilidae). Great Lakes Entomologist 41(3–4): 200-203

[B10] USDA NRCS (2014). The PLANTS Database (http://plants.usda.gov, 24 March 2014). National Plant Data Team, Greensboro, NC 27401-4901 USA

[B12] Grigarick AA Stange LA (1968) The pollen-collecting bees of the Anthidiini of California. Bulletin of the California Insect Survey 9: 1-113

[B13] Miller SR Gaebel R Mitchell RJ Arduser M (2002) Occurrence of two species of Old World bees, *Anthidium manicatum* and *A. oblongatum* (Apoidea: Megachilidae), in northern Ohio and southern Michigan. The Great Lakes Entomologist 35(1): 65-69

[B14] Maier CT (2009) New distributional records of three alien species of Megachilidae (Hymenoptera) from Connecticut and nearby states. Proceedings of the Entomological Society of Washington 111: 775-784. doi: 10.4289/0013-8797-111.4.775

[B15] Tonietto RK Ascher JS (2008) Occurrence of the old world bee species *Hylaeus hyalinatus*, *Anthidium manicatum*, *A. oblongatum*, and *Megachile sculpturalis*, and the native species *Coelioxys banksi*, *Lasioglossum michiganense*, and *L. zophops* in Illinois (Hymenoptera: Apoidea: Colletidae, Halictidae, Megachilidae). Great Lakes Entomologist 41(3–4): 200-203

[B17] Gonzalez VH Griswold T (2013) Wool carder bees of the genus *Anthidium* in the Western Hemisphere (Hymenoptera: Megachilidae): diversity, host plant associations, phylogeny, and biogeography. Zoological Journal of the Linnean Society 168: 221-425. doi: 10.1111/zoj.12017

